# How Microgravity Affects the Biology of Living Systems

**DOI:** 10.1155/2015/863075

**Published:** 2015-01-15

**Authors:** Mariano Bizzarri, Monica Monici, Jack J. W. A. van Loon

**Affiliations:** ^1^Department of Experimental Medicine, Systems Biology Group, University La Sapienza, 00161 Rome, Italy; ^2^ASAcampus Joint Laboratory, ASA Research Division, Department of Experimental and Clinical Biomedical Sciences, University of Florence, 50121 Florence, Italy; ^3^Department of Oral and Maxillofacial Surgery/Oral Pathology, VU-University Medical Center, 1081 HZ Amsterdam, Netherlands

Gravity has constantly influenced both physical and biological phenomena throughout Earth's history. The gravitational field has played a major role in shaping evolution when life moved from water to land, even if, for a while, it has been generally deemed to influence natural selection only by limiting the range of acceptable body sizes, according to Galilei's principle. Indeed, to counteract gravity, living organisms would need to develop systems to provide cell membrane rigidity, fluid flow regulation, and appropriate structural support for locomotion. However, gravity may influence in a more deep and subtle fashion the way the cells behave and build themselves.

The first empirical experiments, mostly done by Russian scientists in the 60s, were unable to unravel major changes after exposure to microgravity, thus nurturing the false notion for which near weightlessness does not get any appreciable effects on living organisms [1, 2]. However, as fundamental investigations began in the space environment, it became evident that biological properties change as gravitational force is diminished, underscoring the relationship between physical force and biological function. Cells exposed to microgravity can indeed be profoundly affected by the physical changes that occur in this unique environment, which include the loss of gravity-dependent convection, negligible hydrodynamic shear, and lack of sedimentation [3–5]. Cell-substrate adhesions, as well as cell-to-cell junctions, are consequently profoundly affected at Earth's gravity, impairing multicellular aggregates and tissue formation, while such structures can be more easily sustained for days or months in microgravity [6]. These modifications eventually lead to a significant change in the way the cell mechanosensor apparatus responds to a wide array of environmental and internal biophysical stresses [7]. As a consequence, enzymatic, genetic, and epigenetic pathways change in concert, leading to several modifications in cells and tissues shape, function, and behavior [8, 9]. Fruitful insights about the involvement of several molecular pathways during microgravity exposure are reported in this issue by the studies of V. Gasperi et al. (unravelling new pathways involved in immune function impairment during spaceflight) and E. Albi et al. (overexpression of Galectin-3 in thyroid follicles due to microgravity-induced membrane remodelling). Namely, a sophisticated analysis of mRNA expression in human blood lymphocytes, carried out by C. Girardi et al., confirmed that microgravity induces a generalized inhibition of proliferation and a contemporary increase in apoptosis rate.

Indeed—and unfortunately—near weightlessness dramatically impairs biological functions and thereby, contrary to what was previously thought [2], cells cannot be considered “blind” with respect to gravity.

The microgravity space environment may result in a challenging threat for living beings, as aptly documented by the paper from C. Nislow et al., showing that spaceflight has subtle but significant effects on core cellular processes including growth control via RNA and ribosomal biogenesis, metabolism, modification, and decay pathways. It is noteworthy that, despite the fact that some reference-genes remain stable during microgravity exposure, several others, investigated in the study of C. S. Thiel et al., change quite dramatically, thus reinforcing the concept that exposure to near weightlessness may have a profound impact on living processes. Namely, it seems that genes involved in ROS detoxification are especially impaired in such condition, as reported by the paper from S. Fengler et al., therefore suggesting how relevant could be the role sustained by the redox status in counteracting at least some downstream consequences of microgravity. Yet, as reported in the article of S. Mugnai et al., both nitric oxide and ROS are likely to play a previously unrecognized role as messengers during the gravitropic response in many root tips. Relevance of oxidative processes during microgravity exposure was also reported by the study of A. M. Rizzo et al., in which a significant increase in oxidative stress has been observed in tardigrades exposed to spaceflight.

Cells may “sense” changes in the microgravitational field through (a) an indirect mechanism (mainly based on the modification of physical properties of their microenvironment); (b) the development of specialized structures for the mechanical perception and transduction of gravitational forces (like the cytoskeleton); and (c) changes in the dynamics of enzymes kinetics or protein network self-assembly. It is worth noting that the latter two processes are dramatically affected by nonequilibrium dynamics. Nonlinear dynamical processes far from equilibrium involve an appropriate combination of reaction and diffusion, and the pattern arising from those interactions is tightly influenced by even minimal changes in reactant concentrations or modification in the strength of the morphogenetic field [10]. Processes of this kind are called Turing or dissipative structures, given that a consumption of energy is required to drive and maintain the system far from equilibrium. That prerequisite is needed in order to allow the system to promptly change its configuration, according to the system's needs. In turn, the dissipative energy provides the thermodynamic driving force for the self-organization processes. Some experimental evidence has already been provided that change of the gravitational field may significantly affect some nonlinear reactions occurring within cells and tissues [11, 12]. Herein, a further confirmation is provided by the article of M. G. Masiello et al., in which the near weightlessness condition is shown to drive the systems towards different attractor states, thus enabling cells to acquire new and unexpected phenotypes in the course of a true phase transition [13]. According to such results, gravity seems to be an “inescapable” constraint that obliges living beings to adopt only a few configurations among many others. By “removing” the gravitational field, living structures will be free to recover more degrees of freedom, thus acquiring new phenotypes and new functions/properties. That statement raises several crucial questions. Some of these entail fundamentals of theoretical biology, as they question the gene-centered paradigm, according to which biological behavior can be explained by solely genetic mechanisms [14].

What are the mechanism(s) through which microgravity may so profoundly modify cell function and structure? Several studies included in this issue deal with that topic, calling into the question the pivotal role sustained by the cytoskeleton in mediating several microgravity-based effects.

A common outcome in nearly all cell types exposed to microgravity is indeed the alteration of cytoskeletal elements: actin, microfilaments, and microtubules [15, 16]. Disorganization of basic cellular architecture can affect activities ranging from cell signalling and migration to cell cycling and apoptosis. In this issue, K. Paulsen and colleagues investigated how surface expression of ICAM-1 protein and expression of ICAM-1 mRNA in cells of the monocyte/macrophage system change in microgravity. Given that ICAM proteins are essential for cell-to-cell adhesion as well as for cytoskeleton proper functioning, such results outline the involvement of the cytoskeleton system in mediating at least some effects due to microgravity. That statement is further reinforced by the paper from F. Louis et al. in which dramatic decrease in RhoGTPases activity has been documented. RhoGTPases represent a unique hub for integration of biochemical and mechanical signals. As such, they are probably very rapidly involved in a cell's adaptation to microgravity-related conditions. Additionally, RhoGTPases activity is tightly and mechanistically bound to alterations of the cytoskeleton, adhesion, and fibrillogenesis as well as to an enhancement of ROS delivery. As a result, RhoGTPases may be considered true mechanosensitive switches responsible for cytoskeletal dynamics and cells commitment. Relevant modification of the cytoskeleton architecture and microtubule organization in testicular cells has been also reported in the study by F. Ferranti et al., where a significant correlation between cytoskeleton abnormalities induced by simulated microgravity and enhanced autophagy was recorded. Yet, cytoskeleton changes affect different cell types, including endothelial cells. In the paper of J. Maier et al., it is shown that endothelial cells are highly sensitive to gravitational stress, as microgravity leads to changes in the production and expression of vasoactive and inflammatory mediators and adhesion molecules, which mainly results from changes in the remodelling of the cytoskeleton and the distribution of* caveolae.* In addition, by keeping in mind that the cytoskeleton dynamics is a fundamental player in cell proliferation and migration, it is not surprising that microgravity significantly affects the flytrap closure, a process involving not only the actin dynamics but also the ion channels and aquaporin activities, as evidenced in the article from C. Pandolfi et al.

Cytoskeleton changes have also profound consequences on both cell shape and tissue modelling. Simulated near weightlessness in human volunteers is associated with a significant change in arterial geometry, flow, stiffness, and shear rate as documented by C. Palombo et al. Microgravity is acting on endothelial cells also through modulation of P2-receptor and the release of several cytokines, as reported by the study from Y. Zhang et al. Given that P2-receptor artificial ligands are applied as drugs, it is reasonable to assume that they might be promising candidates against the cardiovascular deconditioning the astronauts experience during spaceflight.

Overall the alterations occurring in microgravity have undoubtedly significant backwashes on the physiological homeostasis of the whole organism. Such aspect is highlighted by two papers from the group of F. Lacquaniti et al. dealing with the effects of near weightlessness on nervous system function. Gravity is indeed crucial for spatial perception, postural equilibrium, and movement generation. The brain may deal with the gravitational field by integrating a wide array of different signals, thus enabling the system to trigger the most appropriate response. F. Lacquaniti et al. provide compelling evidence that this ability depends on the fact that gravity effects are stored in brain regions which integrate visual, vestibular, and neck proprioceptive signals, where the nervous system combines this information with an internal model of gravity effects. The second study evidenced in turn the beneficial effect of the neurophysiologic adaptation to near weightlessness and how knowledge acquired on this field may even enhance the development of innovative technologies for gait rehabilitation.

Research on microgravity and hypergravity effectively advances our knowledge on physiology and biochemistry, thus providing valuable data and models for the understanding for some important human diseases. Moreover, space-based research has played and presumably will continuously play an important role in reformulating the theoretical framework in biology and physiology and may serve as a novel paradigm for innovation. Namely, microgravity-related research fostered the development of new tools-like for culturing cells in three dimensions. It is now well understood that 3D growth environments that facilitate unrestricted cell-cell interactions are mandatory for defining the biology of cancer cells and tissues, including tumour formation, tumour microenvironment, and tumour progression [17, 18]. Indeed, three-dimensional culture in real and simulated microgravity allows a more precise appreciation of the role the biophysical constraints play in shaping cell phenotypes and functions. In turn, such devices may help in improving tissue-engineering techniques. Experimental models of cells/tissues cultures in both simulated and real microgravity need, however, to be further improved in order to obtain more reliable and reproducible data and to minimize the impact of confounding factors. Such studies may indeed provide valuable information about modulations in signal transduction, cell adhesion, or extracellular matrix induced by altered gravity conditions. These systems also facilitate the analysis of the impact of growth factors, hormones, or drugs on these tissue-like constructs in order to better address issues like pharmacokinetics and pharmacodynamics. Paradigmatic examples of such studies are reported in this issue by the articles of several groups (C. Ulbrich et al.; C. Morabito et al.; V. Gupta et al.), some of which (S. L. Wuest et al.) critically reviewed the reliability of available technical tools (like the Random Positioning Machine). These facilities may also allow investigating developmental and organogenesis processes.

The motivation for this focussed issue of the Biomed Research International Journal is to take stock of the state of research and identify possible areas for future development. There is an urgent need for this, as the last comprehensive collection of studies devoted to space biomedicine research dates back to the 90s [19].

As editors we have collected an eclectic mix of articles, provided by research groups fully involved in space biomedicine research and actively participating in studies carried out both on the International Space Station and on the ground, by means of different techniques enabling performing conditions of simulated near weightlessness and increased gravity. This is not a “one view fits all” approach. It is rather one to “let a hundred flowers bloom.” Yet, they provide a fruitful overview on what is going to come from space biomedicine research. Overall, studies reported in the issue demonstrated how relevant physical cues may be in shaping biological phenotypes and function, influencing so in depth molecular and genetic pathways. It is regrettable to notice that such influences have been for so long overlooked by the scientific mainstream [20, 21]. Furthermore, microgravity studies forced us to develop new technological solutions and more appropriate experimental models. Thereby, knowledge gathered in space research has offered an invaluable support in understanding both human physiology and pathology, fostering technological innovation and the development of priceless medical and experimental devices.

This is why it has been argued that the ultimate reason for human space exploration is precisely to enable us to discover ourselves. Undoubtedly, the microgravity and space related research present an unlimited horizon for investigation and discovery. Controlled studies conducted in microgravity can advance our knowledge, providing amazing and unforeseen insights into the biological mechanism underlying physiology as well as many relevant diseases like cancer [22].

*Mariano Bizzarri* *Monica Monici* *Jack J. W. A. van Loon*

## References

[1] Montgomery P. O., Cook J. E., Reynolds R. C. (1978). The response of single human cells to zero gravity. *In Vitro*.

[2] Tairbekov M. G., Parfyonov G. P., Shepelev E. Y., Sushkov F. V. (1983). Experimental and theoretical analysis of the influence of gravity at the cellular level: a review. *Advances in Space Research*.

[3] van Loon J. J. W., Brinckmann E. (2007). The gravity environment in Space experiments. *Biology in Space and Life on Earth. Effects of Spaceflight on Biological Systems*.

[4] Hammond T. G., Hammond J. M. (2001). Optimized suspension culture: the rotating-wall vessel. *American Journal of Physiology—Renal Physiology*.

[5] Todd P. (1989). Gravity-dependent phenomena at the scale of the single cell. *ASGSB Bulletin*.

[6] Freed L. E., Langer R., Martin I., Pellis N. R., Vunjak-Novakovic G. (1997). Tissue engineering of cartilage in space. *Proceedings of the National Academy of Sciences of the United States of America*.

[7] Klein-Nulend J., Bacabac R. G., Veldhuijzen J. P., Van Loon J. J. W. A. (2003). Microgravity and bone cell mechanosensitivity. *Advances in Space Research*.

[8] Pardo S. J., Patel M. J., Sykes M. C. (2005). Simulated microgravity using the Random Positioning Machine inhibits differentiation and alters gene expression profiles of 2T3 preosteoblasts. *American Journal of Physiology—Cell Physiology*.

[9] Monici M., Fusi F., Paglierani M. (2006). Modeled gravitational unloading triggers differentiation and apoptosis in preosteoclastic cells. *Journal of Cellular Biochemistry*.

[10] Nicolis G., Prigogine I. (1977). Introduction. *Self-Organization in Nonequilibrium Systems: From Dissipative Structures to Order Through Fluctuations*.

[11] Stiles P. J., Fletcher D. F. (2001). The effect of gravity on the rate of a simple liquid-state reaction in a small, unstirred cylindrical vessel. *Physical Chemistry Chemical Physics*.

[12] Papaseit C., Pochon N., Tabony J. (2000). Microtubule self-organization is gravity-dependent. *Proceedings of the National Academy of Sciences of the United States of America*.

[13] Bizzarri M., Giuliani A., Dehmer M., Emmert-Streib F., Graber A., Salvador A. (2011). Represeenting cancer cell trajectories in a phase-space diagram: switching cellular states by biological phase transitions. *Applied Statistics for Network Biology: Methods in Systems Biology*.

[14] Bizzarri M., Cucina A., Palombo A., Grazia Masiello M. (2014). Gravity sensing by cells: Mechanisms and theoretical grounds. *Rendiconti Lincei*.

[15] Lewis M. L. (2004). The cytoskeleton in spaceflown cells: an overview. *Gravitational and Space Biology Bulletin*.

[16] Vorselen D., Roos W. H., MacKintosh F. C., Wuite G. J. L., van Loon J. J. W. A. (2014). The role of the cytoskeleton in sensing changes in gravity by nonspecialized cells. *FASEB Journal*.

[17] Grimm D., Wehland M., Pietsch J. (2014). Growing tissues in real and simulated microgravity: new methods for tissue engineering. *Tissue Engineering Part B: Reviews*.

[18] Souza G. R., Molina J. R., Raphael R. M. (2010). Three-dimensional tissue culture based on magnetic cell levitation. *Nature Nanotechnology*.

[19] Schmitt D. (1999). Workshop purpose and structure. *The FASEB Journal*.

[20] Bizzarri M., Pasqualato A., Cucina A., Pasta V. (2013). Physical forces and non linear dynamics mould fractal cell shape. Quantitative morphological parameters and cell phenotype. *Histology and Histopathology*.

[21] Monici M., van Loon J. (2010). *Cell Mechanochemistry. Biological Systems and Factors Inducing Mechanical Stress, Such as Light, Pressure and Gravity*.

[22] Becker J. L., Souza G. R. (2013). Using space-based investigations to inform cancer research on Earth. *Nature Reviews Cancer*.

